# Molecular Insights into mRNA Polyadenylation and Deadenylation

**DOI:** 10.3390/ijms231910985

**Published:** 2022-09-20

**Authors:** Junjie Liu, Xubing Lu, Siyu Zhang, Ling Yuan, Yadong Sun

**Affiliations:** School of Life Science and Technology, ShanghaiTech University, Shanghai 201210, China

**Keywords:** poly(A) tail, polyadenylation, deadenylation, 3′-end processing, RBBP6/Mpe1, PAN2-PAN3, CCR4-NOT, PABPC, RNA-binding protein

## Abstract

Poly(A) tails are present on almost all eukaryotic mRNAs, and play critical roles in mRNA stability, nuclear export, and translation efficiency. The biosynthesis and shortening of a poly(A) tail are regulated by large multiprotein complexes. However, the molecular mechanisms of these protein machineries still remain unclear. Recent studies regarding the structural and biochemical characteristics of those protein complexes have shed light on the potential mechanisms of polyadenylation and deadenylation. This review summarizes the recent structural studies on pre-mRNA 3′-end processing complexes that initiate the polyadenylation and discusses the similarities and differences between yeast and human machineries. Specifically, we highlight recent biochemical efforts in the reconstitution of the active human canonical pre-mRNA 3′-end processing systems, as well as the roles of RBBP6/Mpe1 in activating the entire machinery. We also describe how poly(A) tails are removed by the PAN2-PAN3 and CCR4-NOT deadenylation complexes and discuss the emerging role of the cytoplasmic poly(A)-binding protein (PABPC) in promoting deadenylation. Together, these recent discoveries show that the dynamic features of these machineries play important roles in regulating polyadenylation and deadenylation.

## 1. Introduction

Poly(A) tails, as a major modification of mRNA, are present on almost all eukaryotic mRNAs. The accurate formation of 3′ end on newly synthesized mRNA, known as polyadenylation, is critical for mRNA maturation, mRNA stability, and mRNA export from the nucleus into the cytoplasm. Poly(A) tails are bound by the poly(A)-binding protein (PAB or PABP). The nuclear isoform PABPN1 is essential for the efficient polymerization of poly(A) tails [1,2,3] and is substantially correlated with poly(A) tail length [4]. In the cytoplasm, PABPN1 is replaced by cytosolic poly(A)-binding protein (PABPC), which protects mRNA from non-specific degradation and promotes mRNA translation. Deadenylation, a process that can release PABPC and initiate mRNA decay, is controlled by a variety of deadenylases to dynamically modulate poly(A) tail length. Poly(A) tails of various lengths were discovered on mRNAs in the early 1970s [5,6]. The length of the poly(A) tail has been found to be closely related to almost every stage in the mRNA life cycle, including transcription termination, mRNA subcellular localization, mRNA quality control, translation efficiency, and mRNA decay [7,8,9]. Poly(A) tails in mammals can be up to 200–250 nt [6] and 90 nt long in yeast [10]. Mutations and defects in these two key processes, polyadenylation and deadenylation, will result in human diseases and viral infections [11,12,13].

**Figure 1:**
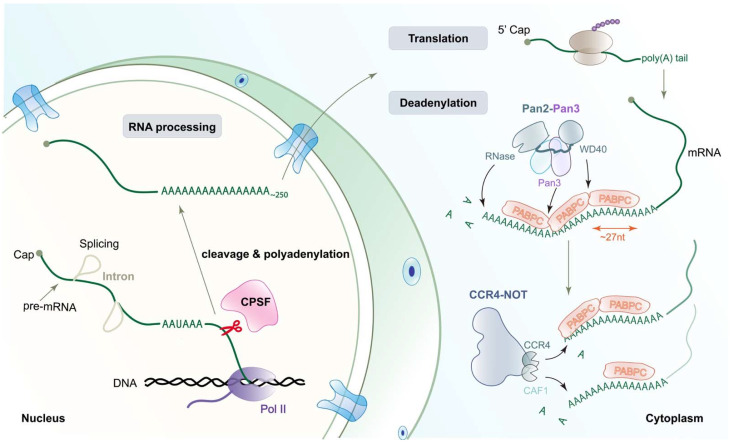
Schematic representation of polyadenylation and deadenylation. In the eukaryotic nucleus, primary RNA transcripts undergo extensive modifications to generate mature functional mRNA; this process is named RNA processing, which includes 5′ capping, splicing, and 3′ polyadenylation. It must be noted that 3′-end processing includes two steps, cleavage at a specific site (the scissors, indicating the site of cleavage by CPSF) and addition of a poly(A) tail at the same site. Then, the mature mRNAs with long poly(A) tails will be exported to the cytoplasm and translated into protein. PABPC covers the poly(A) tail, protecting mRNA from non-specific degradation and meanwhile promoting poly(A) tail removal or shortening via interactions with PAN2-PAN3 and CCR4-NOT complexes (deadenylation). Pan3 and the WD40 domain in Pan2 recognize the interface between different PABP molecules. Deadenylase CCR4 works on PABPC-bound A tails, whereas CAF1 only trims naked poly(A) RNA. CPSF, cleavage and polyadenylation specificity factor.

The nascent pre-mRNA is polyadenylated in the following two steps: cleavage and polyadenylation (Figure 1). The molecular insight into poly(A) tail biosynthesis has been revealed by the earlier crystal structure of yeast Pap1 in complex with poly(A) RNA [14]. In charge of mRNA cleavage is the conserved endonuclease CPSF73 [15], which has recently been identified as a potential therapeutic target for several cancers, inflammatory diseases, and protozoan infections [16,17,18,19]. Deadenylation, on the other hand, contributes to the initiation of mRNA degradation and serves as a checkpoint for gene expression [20]. Both polyadenylation and deadenylation are carried out by large protein machineries, consisting of a great number of conserved and highly regulated multiprotein sub-complexes. How these sub-complexes interact with each other in a cooperative manner and, consequently, safeguard the accuracy of these two important processes remains elusive. With the aid of recent breakthroughs in sequencing methods and cryo-electron microscopy (cryo-EM) techniques, our knowledge of molecular insights into polyadenylation and deadenylation has been expanded.

In this review, we outline current structural investigations into the human and yeast pre-mRNA 3′-end processing complexes, focusing on their recognition of the polyadenylation signal (PAS), machinery organization, and cleavage activation. In particular, we highlight the reconstitution of the active human canonical pre-mRNA 3′-end processing systems, which clarifies, for the first time, the minimal composition of human machineries, and address the functions of RBBP6/Mpe1 in activating the entire machinery. We also describe recent molecular insights into the PAN2-PAN3 and CCR4-NOT deadenylation complexes and discuss the role of cytoplasmic PABPC in promoting deadenylation. Finally, we offer an outlook for future research.

## 2. The Cleavage Step in Polyadenylation

Pre-mRNA 3′-end processing includes endonuclease-mediated cleavage of the pre-mRNA at a specific site and poly (A) polymerase-mediated insertion of a poly(A) tail. The only known exception is replication-dependent histone pre-mRNAs in metazoan, which only involves the cleavage step [21,22]. The cleavage step in polyadenylation is carried out by a series of conserved multiprotein complexes, which were first accomplished in cell extracts in the 1980s [23,24]. During nearly four decades of research, several groups have made tremendous efforts to reconstitute this biological process with recombinant proteins in vitro, which not only simplifies the biochemical reaction for studying the mechanism of the whole machinery and its regulatory factors, but also provides an easily accessible system for identifying new small-molecule inhibitors against CPSF73 [25,26]. Recent studies have defined a minimal list of essential components for this process through the successful reconstitution of pre-mRNA 3′-end processing machinery in vitro [27,28,29,30]. By combining the new biochemical and structural studies, the composition and molecular mechanism for pre-mRNA 3′-end cleavage are highly conserved between yeast and humans.

### 2.1. Essential Components in Canonical Pre-mRNA 3′-End Processing Machinery

The essential components of human canonical machinery include CPSF (cleavage and polyadenylation specificity factor), CstF (cleavage stimulation factor), CF IIm (cleavage factor IIm), PAP(poly (A) polymerase) and Rbbp6 (retinoblastoma-binding protein 6) [27,29]. Surprisingly, with the exception of symplekin, the yeast homologs of all the essential factors involved in human canonical machinery are necessary for yeast pre-mRNA 3′-end processing machinery as well [31], illustrating that 3′-end processing is highly conserved in eukaryotes. The detailed composition of each factor in humans and yeast is shown in Table 1. Specifically, earlier studies indicated that human RBBP6 is involved in human pre-mRNA 3′-end processing, but omitted or underestimated in previous reconstitution and structural studies, due to the fact that RBBP6 is not connected with human CPSF in a stable manner [32,33]. In contrast, yeast Mpe1 is a stable subunit of CPF and binds to Ysh1 (human homolog CPSF73) [28]. The most recent structural and biochemical studies demonstrate the essential role of RBBP6/Mpe1 in regulating 3′-end cleavage, polyadenylation, and transcription termination [27,29,34]. CF Im (cleavage factor Im), a sub-complex only found in multicellular eukaryotes, is dispensable in pre-mRNA 3′-end processing. However, CF Im plays a regulatory role in alternative polyadenylation (APA) by specifically binding to an upstream UGUA motif, altering the efficiency of different poly(A) sites and generating distinct mRNA 3′UTR, which is particularly important for gene regulation [35,36]. The essential components of human histone pre-mRNA 3′-end processing have also recently been defined by the reconstitution and structural studies, which include the histone pre-mRNA cleavage complex (HCC), the stem loop binding protein (SLBP), the U7 small nuclear ribonucleoprotein (snRNP), and FLASH [30]. HCC contains CPSF73, CPSF100, symplekin, and CstF64. The molecular insights into HCC may be similar to its equivalent mCF (mammalian cleavage factor, consisting of CPSF73, CPSF100, and symplekin) in canonical macheniry, as discussed in more detail below.

### 2.2. CPSF160-WDR33 Complex Acts as a Rigid Interaction Platform

**Figure 2:**
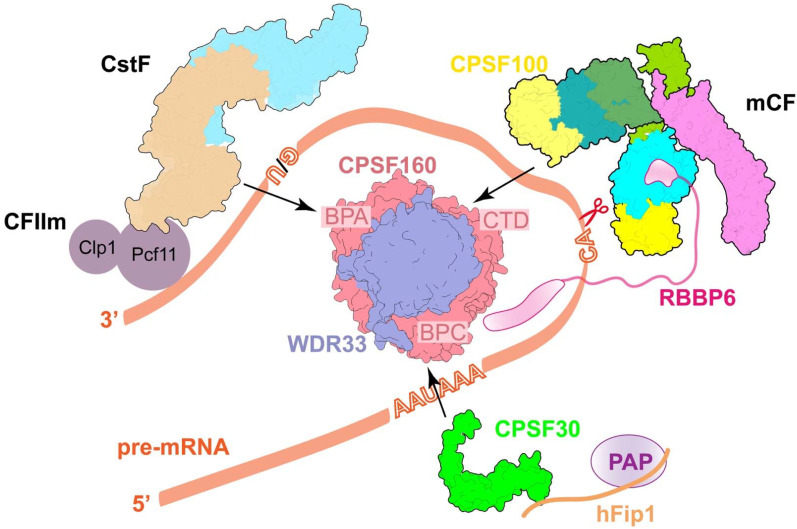
Schematic drawing of the minimal mammalian canonical 3′-end processing machinery. The organizations of the CPSF160-WDR33, mCF, CPSF30, and CstF complex are based on recent structural studies [34,37,41,42,43]. This view of the CPSF160-WDR33 complex is from the bottom, showing that the CPSF160-WDR33 complex functions as a core of the machinery to recruit CPSF30, CPSF100, and CstF77 to the BPC, CTD, and BPA regions of CPSF160, respectively. All of these bindings require both CPSF160 and WDR33. CPSF30 and WDR33 take part in PAS signal (AAUAAA) recognition. PAP is recruited by the CPSF30-hFip1 complex. Based on the high sequence conservation that exists between RBBP6 and yeast Mpe1, it is possible that RBBP6 binds to the metallo-β-lactamase domain of CPSF73 and meanwhile connects to the mPSF module. CTD, C-terminal domain.

CPSF160, as a scaffold protein, contains three β-propellers (BPA, BPB, and BPC) and their relative positions are stabilized by the WD40 domain in WDR33. As a result, CPSF160 and WDR33 form a tight heterodimeric subcomplex that functions as a rigid interaction platform of the canonical machinery and recruits other factors to its distinct areas (Figure 2) [37]. The molecular architecture of human 3′-end processing machinery shows that CPSF30, CPSF100, and CstF77, respectively, bind to the BPC, CTD, and BPA regions of CPSF160, making contact with both CPSF160 and WDR33 (Figure 2) [37]. CPSF30 takes part in RNA recognition and recruits hFip1, and thereby PAP. CPSF100 forms a heterodimer with CPSF73 in mCF for pre-mRNA cleavage. CstF77 is in a complex with CstF50 and CstF64 is used for the recognition of downstream elements (DSE). CPSF30-hFip1-PAP, mCF, and CstF are all highly dynamic [37,38]. They assemble with the CPSF160-WDR33 core, waiting for additional factors and pre-mRNA substrates to initiate the RNA cleavage activity. These are described in further depth in the following paragraphs. Similar interactions were also observed in the yeast homologs, Yth1 (CPSF30) and Cft2 (CPSF100) to Pfs2 (WDR33) and Cft1 (CPSF160). Unexpectedly, recent structural and biochemical studies have shown that Mpe1 makes multiple contacts with Pfs2 and Cft1 as well [34]. Interestingly, the Cft2 protein itself somehow interferes with the binding of Mpe1 to the yeast polymerase module [34], indicating that machinery assembly is a highly regulated event that may be a kind of safeguard mechanism. More research is needed to figure out the exact mechanism. On the other hand, the overall structure of the CPSF160-WDR33 complex is similar to that of the DDB1-DDB2 DNA repair complex [39]. All three β-propellers that are present in DDB1 offer a surface for interactions with other factors [40], while the binding partner for the BPB domain of CPSF160 remains unknown.

### 2.3. The Recognition of PAS

**Figure 3:**
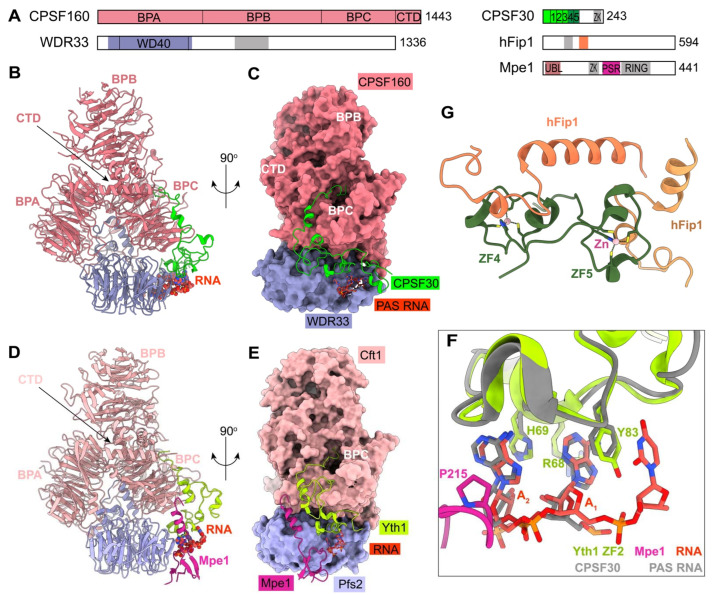
Molecular architecture of human mPSF and the polymerase module of yeast CPF. (**A**) Domain organizations of the subunits of mPSF, and Mpe1. The collagen-like domain in WDR33 is in gray. The zinc fingers (ZFs) of CPSF30 are in green and dark green, and the zinc knuckle in gray. The PAP-binding site of hFip1 is in gray, and the CPSF30-binding site in coral/sandy brown. The linker between CPSF30 and PAP binding sites is the central low-complexity region (LCR) in hFip1. The zinc knuckle and ring domain of yeast Mpe1 are in gray. ZK, zinc knuckle; UBL, ubiquitin-like; PSR, pre-mRNA-sensing region. (**B**,**C**) Structure of the human CPSF160-WDR33-CPSF30-PAS RNA complex (PDB 6DNH). The arrow points to the location of the CTD of CPSF160. The PAS RNA is shown in red orange and ball-and-stick model. The structural model in panel C is viewed after a 90° rotation around the vertical axis. The CPSF160 and WDR33 are shown as molecular surfaces. (**D**,**E**) Structure of the yeast Cft1-Pfs2-Yth1-Mpe1-RNA complex (PDB 7ZGP). The domains in the yeast polymerase module are given lighter colors compared to their homologs in human mPSF. The PSR domain of Mpe1 interacts directly with the polymerase module. (**F**) Comparison of the recognition mechanism of A1A2 in the human mPSF (in gray) and yeast polymerase module-Mpe1 complex (in color). The residue P215 of yeast Mpe1 directly contacts with A2. (**G**) Structure of the human CPSF30-hFip1 complex. The ZF4 and ZF5 of CPSF30 are in dark green. The two hFip1 molecules are in coral and sandy brown. Structure figures are produced with ChimeraX [50].

The most common PAS sequence in mammalian pre-mRNAs is the highly conserved AAUAAA hexamer, which is present in over 60% of pre-mRNAs [44,45,46] and usually located 10~30 nucleotides upstream of the cleavage site. The molecular mechanism of PAS RNA recognition is well understood by the recent cryo-EM studies [41,42]. Structures of human mPSF with AAUAAA-containing RNA show that both CPSF30 and WDR33 are essential for high-affinity binding of PAS RNA (Figure 3A–C). The S-shaped sugar-phosphate backbone of the AAUAAA motif bends the six bases into three pairs [42]. The A1-A2 pair is specifically recognized by zinc finger 2 (ZF) of CPSF30 through hydrogen bonding interactions, π stacking, and van der Waals interactions, and ZF3 binds to the A4–A5 pair in the same manner. The U3-A6 forms a Hoogsteen base pair and interacts with two conserved phenylalanine residues in WDR33. The whole PAS is covered and stabilized by the N-terminal loop of WDR33 (Figure 3C). More details have been summarized in several review articles [47,48,49].

In contrast, PAS in yeast, also known as the positioning element, is highly degenerate and less well defined with A-rich sequences [51]. The molecular details of recognition of the yeast positioning element AAGAA have been recently reported by structural biological research [34]. The polymerase module of yeast CPF shares structural similarities with human mPSF (Figure 3D,E). Although the polymerase module is in complex with 42-nt long CYC1 RNA, only three nucleotides of PAS can be assigned with well-ordered electron density. Particularly, the A1-A2 is recognized by ZF2 of Yth1 (CPSF30 homolog in yeast) in a way that is very comparable to that of CPSF30, suggesting that the first A-A dinucleotide of PAS is recognized by a conserved mechanism in eukaryotes (Figure 3F).

Surprisingly, besides Yth1/CPSF30 involved in PAS recognition, the structure shows that yeast Mpe1 directly contacts A2 via residue P215 in the pre-mRNA-sensing region (PSR) (Figure 3F) [34]. However, based on the biochemical evidence, Mpe1 PSR does not have any significant effect on the RNA binding affinity of the polymerase module. In contrast, the binding between Mpe1 and the polymerase module is improved by RNA binding. In addition, RNA binding induces conformational changes in Mpe1, resulting in a more compact structure [34]. All of the evidence presented above points to the possibility that Mpe1 may perform the role of a PAS motif sensor to verify the substrate entering the whole machinery. The residue P215 of Mpe1 and its surrounding residues are conserved in Mpe1 orthologs [34], suggesting that human RBBP6 may function similarly to Mpe1 in human canonical pre-mRNA 3′-end processing. Meanwhile, compared to the structure of mPSF-RNA, the helix in Mpe1 PSR overlaps with CPSF30 residues 22–34, indicating that the equivalent region in RBBP6 PSR may attach to a different position on mPSF [34]. In further research, it should be determined whether or not RBBP6 is able to sense RNA binding in the same way.

### 2.4. The Role of hFip1

Fip1 recruits PAP onto the machinery via binding to CPSF30. However, hFip1 and the C-terminal region of CPSF30, including ZF4-ZF5 and the zinc knuckle, are disordered in all of the existing structures, due to their flexible features relative to the mPSF core. The recent crystal structure reports that both ZF4 and ZF5 can bind to hFip1 (Figure 3G). Furthermore, the CPSF30-hFip1 complex is unexpectedly formed with 1:2 stoichiometry and further recruits two copies of PAP in vitro [43]. This finding is compatible with earlier mass spectrometry studies, showing that there are two copies of Fip1 and Pap1 in the yeast machinery [52]. The residues at the interface between CPSF30 and hFip1 are highly conserved from yeast to human, suggesting that the binding mode might be conserved in most species [43]. Indeed, the essential role of yeast Fip1 in connecting Yth1 and Pap1 has been illuminated in depth as well [38]. In a recent study, it was found that yeast Fip1 is able to stabilize the Yth1 zinc-finger fold by directly interacting with Yth1, and the region for Yth1 binding is conserved and is the only sequence that has low disorder propensity in Fip1 [38]. The rest of the part in Fip1 is largely unstructured. The linker (the central low-complexity region (LCR) in Fip1) between Yth1 and Pap1 binding sites, in particular, remains disordered, even after being assembled into the entire 3′-end processing machinery [38]. Moreover, the dynamics of the central LCR in Fip1 are important for the high efficiency of pre-mRNA 3′-end cleavage [38]. However, the exact mechanism remains unclear, which needs further investigation.

### 2.5. Molecular Architecture of CPSF

**Figure 4:**
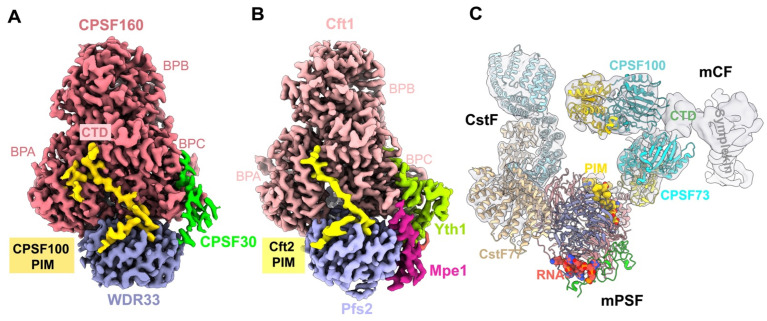
Overall architecture of the human canonical pre-mRNA 3′-end processing machinery. (**A**) Cryo-EM reconstruction of the human mPSF in complex with the CPSF100 PIM (in gold). (**B**) Cryo-EM reconstruction of the yeast polymerase module in complex with the Cft2 PIM (in yellow). (**C**) A model for the mPSF-mCF-CstF complex of the human canonical machinery, which is produced by overlaying the structures of the mPSF-mCF and mPSF-CstF complexes. The view is similar to that in Figure 2. mPSF module is colored as in panel A, and its view is from the bottom. The PAS RNA and PIM are shown as ball-and-stick models. The region of symplekin for CstF64 binding is disordered. The symplekin CTD is labeled in panel C. CstF77 is in blue and beige.

CPSF consists of the following two modules: mPSF that recognizes PAS and recruits PAP to catalyze the polyadenylation as discussed above, and mCF that catalyzes the cleavage reaction [53,54]. mCF contains the endonuclease CPSF73, its homolog CPSF100, and a scaffold symplekin. In contrast to mPSF, the structure of mCF is highly dynamic. CPSF73 and CPSF100 bind to sympekin via their CTDs, generating a trilobal structure, but the relative positions of the three lobes are highly variable [37]. mCF interacts with mPSF, leading to the formation of CPSF. However, CPSF shows structural variability as well. Cryo-EM structures of CPSF revealed that mCF and mPSF tether together via a small peptide (residues 460–486) in CPSF100, named PIM (the PSF interaction motif), which forms extensive hydrophobic interactions within both CPSF160 (BPA and CTD) and WDR33 (Figure 4A) [37]. The PIM is located in a disordered, extremely hydrophilic, and long fragment (~100 residues) inside the β-CASP domain of CPSF100, which explains the highly dynamic nature within CPSF. The sequence in the PIM is highly conserved in CPSF100 homologs, and a similar peptide in yeast Ctf2 (residues 525–562, named yPIM) was also observed by the most recent cryo-EM study [34]. The binding mode of yPIM with Ctf1 and Pfs2 is similar to that with human mPSF (Figure 4B), indicating the conserved role of Ctf2 in tethering the nuclease module to the polymerase module. Interestingly, as previously mentioned, Ctf2 blocks Mpe1 PSR binding to the polymerase module, which can be recovered by adding RNA. However, biochemical data show that Ctf2 and yPIM alone have different effects on the recovery in the presence of RNA, with substoichiometric levels of Mpe1 if Ctf2 is present in the complex [34], suggesting the location of Ctf2 might be regulated in 3′-end processing activation.

Due to the dynamic nature of CPSF and mCF alone, the structure of mCF is limited to a low resolution at 7.4 Å (Figure 4C) [37]. The structures of the catalytic modules of CPSF73 and CPSF100 fit well into two of the three lobes, with the only contacts by their C-terminal domain, suggesting that CPSF73 and CPSF100 form a heterodimer by their CTDs (Figure 4C). However, CPSF73 is still in a close state, since there is no room for RNA to go through its active site, indicating that conformational changes in both mCF and CPSF73 are required for the machinery activation [37]. Symplekin CTD has an elongated shape in the structure, whereas the NTD for CstF64 binding is disordered (Figure 4C) [37].

### 2.6. Accessory Factors CstF and CF IIm

CstF is essential for pre-mRNA 3′-end cleavage as well, but dispensable for the polyadenylation step. It includes three subunits, CstF50, CstF64, and CstF77, but their relative positions are highly variable [37]. Cryo-EM structure analyses show that the bow-shaped HAT-C dimer of CstF77 is recruited to mPSF by interacting with both the BPA domain of CPSF160 and WDR33, and there is no density for CstF50 and CstF64 (Figure 4C), which is consistent with the dynamic nature of CstF and the yeast Rna14-Rna15 complex (homologs of CstF77 and CstF64) [37,55]. The N-terminal RRM of CstF64 is responsible for the recognition of G/U-rich DSE. The C-terminus of CstF77 binds to the CstF64 hinge domain and plays an important role in boosting the RNA binding affinity of RRM [56]. CstF50 only exists in multicellular eukaryotes and has no homolog in yeast. The crystal structure reveals that CstF77 is in a complex with the CstF50 WD40 domain via a segment between its HAT domain and the CstF64 binding site [56]. However, the exact function of CstF50 is still a mystery, although some studies reveal that CstF50 may have a potential role in efficient DSE selection [56].

CF IIm that contains hClp1 and hPcf11 is also essential for cleavage activity, which has been proved by recent biochemical research [57]. Specifically, the 30 FEGP repeats of hPcf11 are critical for RNA cleavage with an unknown function. In yeast, yClp1 and yPcf11, associated with Rna14 and Rna15, together form the factor named CF IA (Table 1), which is essential for yeast machinery as well. However, the FEGP repeats are not conserved in yPcf11. Further structural research is required to figure out the interaction network of CF IIm and clarify its function in eukaryotic pre-mRNA 3′-end processing.

### 2.7. Activation of the Pre-mRNA 3′-End Cleavage

**Figure 5:**
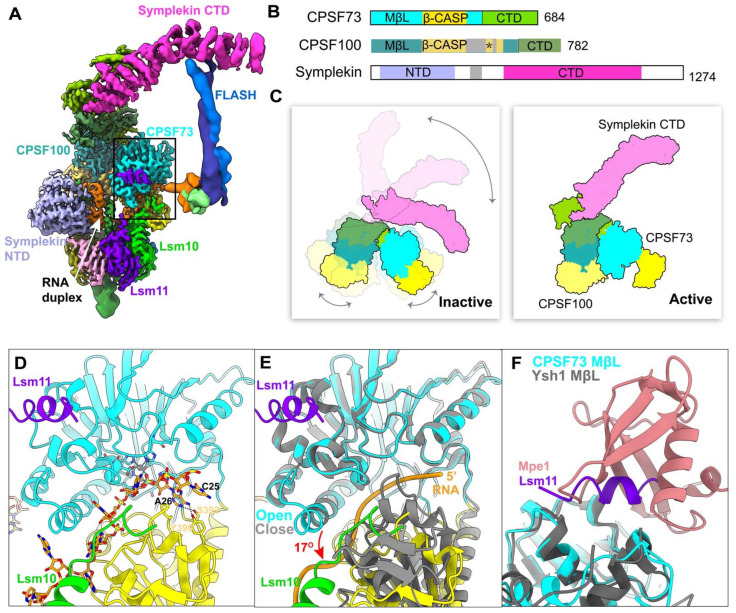
The activation of mCF/HCC and CPSF73. (**A**) Composite cryo-EM density map for the histone pre-mRNA 3′-end processing machinery (reproduced from [30]). The white arrow points to the RNA duplex, showing pre-mRNA in orange and U7 snRNA in green. The positions of CPSF73, CPSF100, symplekin NTD, symplekin CTD, Lsm10, Lsm11, and FLASH are labeled. Specifically, the active CPSF73 is in the black box, and additional details are shown in panels (**D**–**F**). (**B**) Domain organizations of the subunits of mCF. The disordered and highly hydrophilic segment in the β-CASP domain of CPSF100 is in gray, and the position of PIM is labeled as ‘*’(star). (**C**) A schematic drawing of inactive and active mCF. The conformational changes are observed in recent structural studies [30,37]. The proteins are colored as in panel B. After rearrangement, the subunits of mCF become more compact. (**D**) Recognition of the pre-mRNA substrate by CPSF73 (PDB 6V4X). The hydrogen-bond interactions are formed between A26 and the β-CASP domain of CPSF73. (**E**) Overlay of the structure of CPSF73 in the active state (in color, PDB 6V4X) with that of CPSF73 in the inactive state (in gray, PDB 2I7V). The MβL domain was used for the overlay. The β-CASP domain (yellow) makes a rotation of 17° relative to MβL domain (cyan). The positions of Lsm10 and Lsm11 are labeled. RNA is shown by orange ribbons. (**F**) Overlay of the structure of the Lsm11-CPSF73 with Mpe1-Ysh1 complex (PDB 6I1D). The MβL domain was used for the overlay, which is in cyan for CPSF73 and in gray for Ysh1. The binding sites of Mpe1 and Lsm11 on the MβL domain are overlapped. MβL, metallo-β-lactamase.

The overall organization of the human pre-mRNA 3′-end processing machinery shows the highly dynamic nature, which is also observed in yeast machinery and histone pre-mRNA 3′-end processing machinery. The extensive rearrangements of mCF/HCC and CPSF73 for catalyzing the processing are demonstrated by a recent cryo-EM study of histone recombinant machinery (Figure 5A–C), which is the only structure so far that shows the endonuclease CPSF73 in an open, active state, in which CPSF73 captures the pre-mRNA in its active site, ready to be cleaved (Figure 5D) [30]. The adenine base at the cleavage site forms hydrogen bonds with CPSF73 and the phosphate after this adenine is located at the CPSF73 active site, explaining why CPSF73 prefers an adenine as the cleavage site (Figure 5D). The binding mode clearly shows the molecular mechanism for the cleavage reaction. Compared with CPSF73 in a close state, the β-CASP domain makes a 17° rotation relative to the metallo-β-lactamase domain, thus creating a narrow canyon between the two domains, which can only accommodate single-stranded RNA (Figure 5E) [30]. Most importantly, the rearrangement of HCC is induced by their recognition of RNA duplex, which makes symplekin-NTD, CPSF73, and CPSF100 more compact (Figure 5A,C). A new interface is generated by the formation of a CPSF73-CPSF100 pseudo-dimer, which fixes the catalytic module of CPSF73 against the Lsm10 subunit of the U7snRNP. Lsm10 pushes CPSF73 open (Figure 5A). The targeted single-strand RNA can be guided into the canyon by the long handle and further sensed by Lsm10 (Figure 5A). As previously noted, the activation of CPSF73 in histone machinery requires the coordinated assembly of HCC with U7 snRNP and pre-mRNA.

Remarkably, HCC is recruited to histone machinery by two tethering contacts, FLASH with symplekin CTD and a segment of Lsm11 with the metallo-β-lactamase domain of CPSF73 (Figure 5A). The same surface on CPSF73 is also observed in the Ysh1-Mpe1 interaction, which is important for promoting cleavage and polyadenylation in yeast (Figure 5F) [28,34]. In addition to this, the PSR motif in Mpe1 is stably bound to Pfs2 through sensing the PAS RNA/positioning element, which leads to the rearrangement of Mpe1 (Figure 3F). Thus, Mpe1 connects both the nuclease module and the polymerase module through its ubiquitin-like domain (UBL) and PSR, which is conserved in RBBP6 from the sequence alignment analysis [28,34]. Recently, two independent studies discussed the critical role of human RBBP6 in activating mRNA processing. Similar to Mpe1, RBBP6 associates with CPSF as well, but in an RNA-dependent manner. The N-terminal domain of RBBP6, including UBL, zinc knuckle, and PSR motif, is sufficient to activate canonical pre-mRNA 3′-end cleavage [27,29]. The structural and biochemical studies support the argument that RBBP6/Mpe1 may act as an essential activator of eukaryotic canonical machinery by acting as an RNA sensor and, meanwhile, tethering the endonuclease CPSF73/Ysh1 (Figure 2).

Interestingly, symplekin, a component of HCC/mCF, plays multiple important roles in mammalian pre-mRNA 3′-end processing, whereas Pta1 (homolog of symplekin) is dispensable in yeast machinery. According to the analysis of the structure of histone machinery, symplekin NTD is responsible for RNA duplex recognition, leading to HCC rearrangement (Figure 5A). In contrast, symplekin NTD is dispensable in canonical machinery [27], suggesting the mechanism of mCF rearrangement and CPSF73 activation is different in histone and canonical machinery. However, there are some differences in the current findings about the necessity of PAP and ATP in pre-mRNA 3′-end cleavage, for which further studies are needed in order to clarify their criticality.

## 3. The Biphasic Model of Deadenylation

The dynamic metabolism of the poly(A) tail plays a key role in the regulation of gene expression, which in turn directly determines the composition of the cellular proteome and, as a result, affects most life activities in eukaryotes. In the cytoplasm, poly(A) tails are coated by PABPC (PABPC1 in mammals, Pab1 in yeast) and shortened in a 3′-to-5′ direction by two highly conserved multiprotein complexes, PAN2-PAN3 and CCR4-NOT, in a biphasic manner (Figure 1) [58,59,60]. In the initial phase, PAN2-PAN3 is responsible for the slower removal of the distal part of the poly(A) tail (200–110 nt in mammals, 90 nt in yeast), while in the second, fast phase, the CCR4-NOT complex acts mainly on the final 110 nt (in mammals) to a very short one, triggering mRNA decay [61]. The exonucleases (deadenylase) in these two complexes are PAN2, CNOT6/CNOT6L and CNOT7/CNOT8, respectively. Recent insights from biochemical reconstitution and structural biology have brought to light the dichotomous role of PABPC in mRNA life, the organizational principles of yeast Pan2-Pan3 on poly(A) RNP, and the important role that non-enzymatic components play in the CCR4-NOT complex (see below).

### 3.1. PABPC’s Role in Deadenylation

PABPC contains four RNA recognition motif (RRM) domains, which are responsible for binding poly(A) tails, followed by a proline-rich linker and a C-terminal domain. An earlier crystal structure has revealed that the poly(A) tail in an extended conformation is specifically recognized by a long, narrow groove, created by the tandem antiparallel β sheets of RRM1 and RRM2 of human PABPC1 [62]. The RRM1 is located at the 3′ region of the RNA substrate. The sequences of RRM1 and RRM2 are very similar to that of RRM3 and RRM4. However, the binding mode of RRM3 and RRM4 to poly(A) is quite different from that of the tandem RRM1-RRM2 [63,64]. This is possibly due to the length variation in the loop regions between RRMs, which may tightly associate with the RNA-binding affinity and the range of RNA length that one PABPC molecule can protect. PABPC is highly conserved in eukaryotes and both PABPC1 and Pab1 have a RNA-binding footprint of 27 nt [65,66]; thus, a longer poly(A) tail can attract multiple PABPC1 molecules (Figure 1), which are linearly arranged on the tail and form a worm-like structure, as observed in negative-stain studies [67]. PABPC plays a contradictory role in deadenylation. On the one hand, it prevents non-specific degradation of mRNA by specifically binding the poly(A) tail [68], and meanwhile stimulates translation [69]; on the other hand, as discussed below, it promotes poly(A) tail shortening and mRNA decay by interacting with PAN2-PAN3 and CCR4-NOT complexes [60,61,70].

### 3.2. The Architecture and Recognition Mechanism of Poly(A) RNP by Pan2-Pan3 Complex

**Figure 6:**
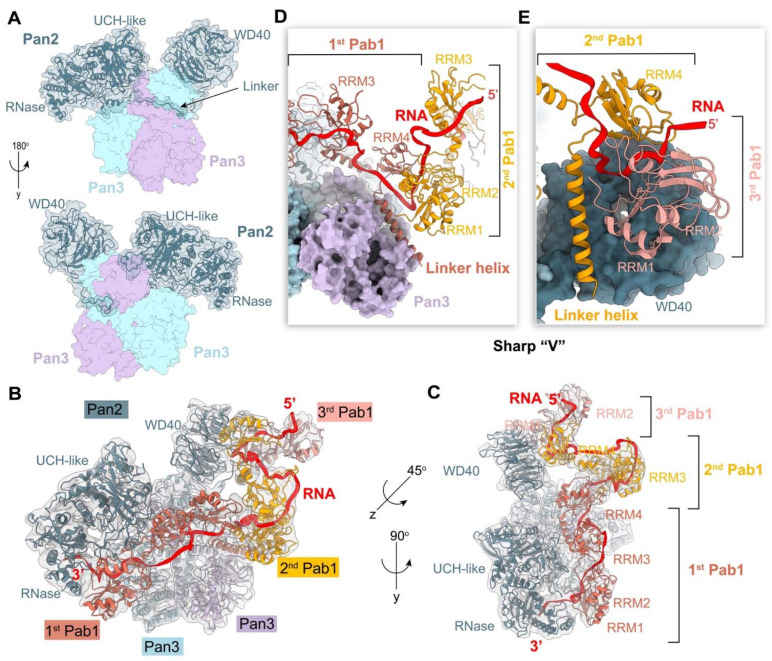
The architecture of the poly(A) RNP-Pan2-Pan3 complex. (**A**) Structure of the apo Pan2-Pan3 complex [60]. The two Pan3 molecules are in light blue and purple, respectively. The molecular surface of Pan2 is shown as a transparent surface. The black arrow indicates the important linker between the UCH domain and the WD40 domain of Pan2. (**B**,**C**) Cryo-EM density for the entire poly(A) RNP-Pan2-Pan3 complex (gray, semitransparent), in side view (**B**) and top view (**C**), fitted with the model of Pab1, Pan2, and Pan3. The poly(A) RNA is shown by a red ribbon. PDB 6R5K. (**D**,**E**) The interaction between the Pan2-Pan3 complex and the Pab1-Pab1 oligomerization interface. The pseudokinase domain of Pan3 (purple) and the WD40 domain of Pan2 are shown on the molecular surface. The linker helix in RRM4 is labeled. The RRM4 and the RRM1-RRM2 domains of the following Pab1 shape the substate RNA into a sharp “V” shape.

PAN2 is a deadenylase, consisting of an N-terminal WD40 domain, an inactive ubiquitin C-terminal hydrolase (UCH), and an exonuclease domain at its C terminus (Figure 6A). PAN2, by forming a complex with PAN3 in a 1:2 stoichiometry (Figure 6A), contributes to the initial trimming of the distal part of the poly(A) tail [71], which requires two or more PABPC1/Pab1 molecules in this process. The recent cryo-EM reconstruction of the yeast Pan2-Pan3 in complex with a poly(A) RNP composed of 90 adenosines and three Pab1 has illuminated the molecular mechanism for why Pan2-Pan3 preferentially acts on a longer poly(A) RNP and how Pab1 stimulates this process (Figure 6) [60]. Based on comprehensively structural and biochemical analyses, it was found that the asymmetric homodimer formed by Pan3 functions as an important interaction hub, recruiting Pan2 via a long linker between the WD40 domain and UCH domain of Pan2, and forming different functional interfaces on opposite sides of the Pan3 homodimer (Figure 6A), which is critical for recognizing the Pab1-90A RNP [60,72]. The Pab1-90A RNP binds to one side of the Pan2-Pan3 complex (Figure 6B,C). The architecture of the poly(A) RNP in this structure interestingly presents repeated arches, which are shaped by RRM3, the Pab1-Pab1 oligomerization interface, and the interacting position with Pan2-Pan3 (Figure 6C–E). The Pab1-Pab1 oligomerization interface adopts a sharp “V” shape, which is formed by a long conserved α-helix (linker helix) from RRM4 and the RRM1 of the following Pab1 molecule (Figure 6D,E). The interface between the first and second Pab1 is recognized by the Pan3 pseudokinase domain (Figure 6D), while the second interface between the second and third Pab1 interacts with the WD40 domain of Pan2 (Figure 6E), indicating that the WD40 domain is the key checkpoint for the length of the poly(A) tail. Moreover, RRM1 of the first instance of Pab1 binding at the most 3′ end drives the poly(A) tail into the active site by interacting with the RNase domain of Pan2. Recent crystal structures show that the deadenylation specificity of Pan2 is determined by recognizing the A-form helical conformation formed by poly(A) RNA, rather than canonical base-specific contacts [73]. In summary, Pab1 stimulates deadenylation by providing the main structural features for the Pan2-Pan3 complex recognition, which is critical for its length specificity.

### 3.3. The Roles of Non-Enzymatic Components in CCR4-NOT Complex

The CCR4-NOT complex is a multi-subunit complex that is partially redundant with PAN2-PAN3, but mainly focuses on removing adenosines proximal to the 3ʹ UTR [59]. Cryo-EM structures of the yeast Ccr4-Not complex have been reported in the past several years [74]. However, the resolution is not sufficient to elucidate the detailed molecular mechanism of the complex assembly and deadenylation.

The CCR4-NOT complex is evolutionarily conserved from yeast to human, but the composition shows species specificity and differs among humans, Drosophila, and yeast [75,76,77,78]. CCR4-NOT in humans contains at least eight subunits, including the following two deadenylases: CNOT6/CNOT6L/CCR4 (also known as Ccr4 in yeast) and CNOT7/CNOT8/CAF1 (also known as Ccr4-associated factor 1, Caf1 in yeast) [79]. Both deadenylases contribute to adenosine removal, but present different activities with PABPC; CCR4 performs its function on PABPC-protected A tails, whereas the activity of CAF1 is blocked by PABPC, resulting in only trimming naked poly(A) RNA and the generation of deadenylation cycles every 27 nt (Figure 1) [61]. However, the molecular basis of the completely opposite effects generated by PABPC with the CCR4-NOT complex remains a mystery. CNOT1 is the largest subunit (>200 kDa) in the complex, acting as an essential scaffold to assemble the CCR4-NOT complex [76], which is roughly divided into the following four modules: C-terminal NOT module (binding to CNOT2 and CNOT3) [80]; catalytic module on the MIF4G domain of CNOT1 (recruiting the two deadenylases) [81]; ubiquitin ligase module next to the catalytic module (binding to CNOT9 and, in some conditions, an E3 ubiquitin ligase, CNOT4) [82,83]; N-terminal module (recruiting CNOT10 and CNOT11). The non-enzymatic components, CNOT10 and CNOT11, are conserved in eukaryotes, except for yeast [76,84,85]. Both CNOT9 and CNOT10:CNOT11 modules bind to RNA directly and stimulate deadenylation [86]. The non-enzymatic modules in the human CCR4-NOT complex play important roles in increasing deadenylation activity and sequence selectivity, probably due to their ability of providing more RNA-binding sites to cooperatively strengthen the interaction between the complex and the RNA substrate [86]. However, so far, only limited fragmentary information is available to explain how these subunits act together and form the CCR4-NOT complex. The recent crystal structures of human CCR4-CAF1 show a similar binding mode to that of *S. cerevisiae*. CCR4 binds to CAF1 via the CCR4 LRR domain, which seems to tether the CCR4 nuclease domain by a flexible linker [87]. Therefore, the distance between the two nucleases is probably regulated in the context of the CCR4-NOT complex, which is observed in the latest structural studies on the human catalytic module (CNOT1(MIF4G)-CAF1-CCR4) [88], illustrating that the position of the nuclease domain of CCR4 may be restricted by the non-enzymatic components in the CCR4-NOT complex. The biochemical data also indicate that CAF1 functions as a tunable enzyme, which is highly sensitive to experimental conditions, such as pH, magnesium, and zinc ions [87].

## 4. Conclusions and Future Perspectives

The catalytic activities of the endonuclease CPSF73/Yth1 in polyadenylation, and the deadenylases CAF1 and CCR4 in deadenylation are quite poor if they function as stand-alone enzymes [15,86]. To achieve full functional activities, they have to be assembled into large machineries, with multiple non-enzymatic components. These components are essential for promoting the subcomplex rearrangement and stabilizing the assembly of the machinery, as well as restricting the flexibility by providing additional RNA-binding sites or coordinating a series of interactions among different factors. As a result, these machineries possess significantly stronger enzymatic activities and improved substrate specificity. The overview of the recent progress in structural and biochemical studies of polyadenylation and deadenylation shows that the dynamic features of the machineries play important regulatory roles. However, many questions about the molecular mechanisms of the whole machinery and the role of each component are still pending. Recent structural studies on histone pre-mRNA processing have shown the binding mode of the active CPSF73 with the substrate RNA, suggesting that the distance from the HDE-U7 duplex is a key factor for defining the cleavage site. However, it remains unclear how the cleavage position in canonical machinery is determined. Based on successful reconstitution with recombinant proteins in vitro, the minimal list of essential components in canonical pre-mRNA 3′-end processing machinery has been defined, which indicates that CF IIm and RBBP6/Mpe1 play critical roles in the cleavage activation step [27,29,34,57]. Nevertheless, further studies are required to reveal more details at the molecular level.

In deadenylation, the PAN2-PAN3 and CCR4-NOT complex have different and partially redundant substrate preferences. Although structural analyses have unveiled why yeast Pan2-Pan3 works on longer poly(A) tails [60], the structural details of the CCR4-NOT complex and how PABPC affects CCR4-NOT at the molecular level remain to be determined. Furthermore, it was thought that long poly(A) tails give better stability to mRNAs, but several very stable transcripts with short poly(A) tails have been recently discovered [89,90]. Therefore, the exact relationship between the length of the poly(A) tail and mRNA stability needs further investigation. On the other hand, the poly(A) tail can be elongated not only in the nucleus, but also in the cytoplasm. Cytoplasmic polyadenylation increases protein expression of dormant mRNAs with short poly(A) tails, thereby regulating the translation of specific mRNAs at various times and locations [91]. Notably, there is an alternative pathway for poly(A) tail 3′-end protection from deadenylation, in which the poly(A) tail forms RNA triple helices with cis-acting U-rich RNA elements [92]. Recent structural studies have revealed that a poly(A) 3′-end binding pocket is formed via a steric mechanism, allowing for unexpected kinds of RNA-RNA interaction [93,94]. Overall, the recent progress provides fresh molecular insights into poly(A) tail biology. More exciting research is needed to figure out how poly(A) tail regulation works at the molecular level.

## Figures and Tables

**Table 1:** Human and yeast mRNA 3′-end processing machinery.

Human			Yeast	
Complex	Subunit	Role	Complex	Subunit
**CPSF**	**mPSF ^1^ including**	**PAS Recognition**	**CPF**	
	CPSF160	Scaffold		Cft1
	WDR33	Scaffold, RNA binding		Pfs2
	CPSF30	Recruits hFip1, RNA binding		Yth1
	hFip1	Binds PAP		Fip1
	**mCF ^2^ including**	**Cleavage**		
	CPSF73	Endonuclease		Ysh1
	CPSF100	(Pseudo-)endonuclease, assembles		Cft2
	Symplekin	Scaffold		Pta1 *
	**RBBP6**	Activates cleavage, RNA binding		Mpe1
	**PAP**	**Poly(A) polymerase**		Pap1
**CstF**	CstF50	Scaffold	**CF** **IA**	n.d.
	CstF64	RNA binding		Rna15
	CstF77	Scaffold, assembles		Rna14
**CF IIm**	hPcf11	Binds Pol II		Pcf11
	hClp1	RNA kinase		Clp1
CF Im *	CFIm68	RNA binding		n.d.
	CFIm25	RNA binding		n.d.

n.d., none detected. *, dispensable for the cleavage of mRNA 3′-end processing machinery. ^1^, mPSF is short for mammalian polyadenylation specificity factor. ^2^, mCF is short for mammalian cleavage factor. The essential complexes or subunits in human canonical machinery are shown in bold.
